# Geographic expansion of the introduced *Aedes albopictus* and other native *Aedes* species in the Democratic Republic of the Congo

**DOI:** 10.1186/s13071-024-06137-4

**Published:** 2024-01-26

**Authors:** Fabien Vulu, Kyoko Futami, Toshihiko Sunahara, Pitshou Mampuya, Thierry L. Bobanga, Dieudonne Mumba Ngoyi, Noboru Minakawa

**Affiliations:** 1 Program for Nurturing Global Leaders in Tropical and Emerging Communicable Diseases, Graduate School of Biomedical Sciences, Nagasaki University, Nagasaki, Japan; 2 Department of Vector Ecology & Environment, Institute of Tropical Medicine, Nagasaki University, Nagasaki, Japan; 3 Department of Tropical Medicine, University of Kinshasa, Kinshasa, Democratic Republic of the Congo; 4 Department of Parasitology, National Institute of Biomedical Research, Kinshasa, Democratic Republic of the Congo

**Keywords:** *Aedes* mosquitoes, Arbovirus vectors, Invasion, Distribution, MaxEnt, DR Congo

## Abstract

**Background:**

*Aedes albopictus* has been reported in several Central African countries, including the Democratic Republic of the Congo (DRC). The establishment of this mosquito species poses a serious threat as a vector of various infectious diseases. Although *Ae. albopictus* has been reported in the western region of the DRC, information about its distribution is still scarce in the country. The aim of this study was to investigate the current nationwide distribution of the invasive *Ae. albopictus*, as well as other native *Aedes* mosquitoes, in the DRC and to identify suitable areas for its future expansion.

**Methods:**

Two entomological surveys were conducted in 2017–2019 and 2022. Based on the occurrence sites of *Ae. albopictus*, important environmental variables were identified. Then, geographical areas suitable for *Ae. albopictus* establishment were determined using the maximum entropy model. The distribution and abundance of *Ae. albopictus* were also compared with those of the major native *Aedes* species.

**Results:**

*Aedes albopictus* was found in the western, northern, central, and eastern regions of the DRC, but it was not found in the southeastern region. The maximum entropy model predicted that most parts of the DRC are suitable for the establishment of this mosquito. The unsuitable areas encompassed the eastern highlands, known for their low temperatures, and the southeastern highlands, which experience both low temperatures and a long dry season. The native *Aedes* species found were *Aedes aegypti*, *Aedes simpsoni*, *Aedes africanus*, and *Aedes vittatus*. *Aedes albopictus* dominated in the western and northern regions, while *Ae. aegypti* was more prevalent in other regions.

**Conclusions:**

*Aedes albopictus* has been well established in the western and northern regions of the DRC. This mosquito is expanding its distribution while replacing the native *Aedes* species. Most of the country is suitable for the establishment of this mosquito species, except the highlands of the eastern and the southeastern regions.

**Graphical Abstract:**

**Figure:**
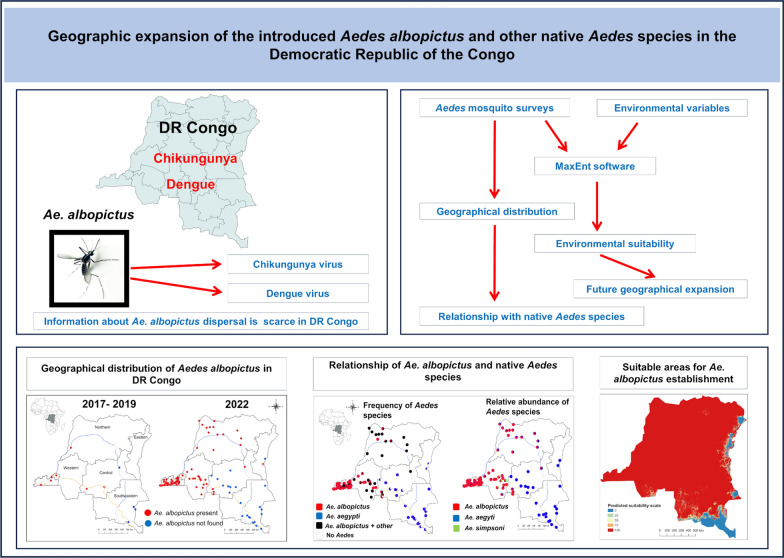

**Supplementary Information:**

The online version contains supplementary material available at 10.1186/s13071-024-06137-4.

## Background

*Aedes albopictus* (Skuse, 1895) (Diptera: Culicidae), known as the Asian tiger mosquito, is the most well-known invasive mosquito to have originated from East Asia [1]. This mosquito has expanded its distribution globally and has become a serious threat to public health because of its ability to vector chikungunya, dengue, and zika viruses [1–4]. In central Africa, this mosquito was first reported from Cameroon in 2000 [5] and subsequently from other countries including the Democratic Republic of the Congo (DRC) [6–10]. Following the mosquito invasion, multiple dengue and chikungunya outbreaks have occurred in central Africa [10–18].

In the DRC, the co-circulation of these viruses as well as the yellow fever virus has posed a serious public health challenge [18, 19]. Successive outbreaks of these vector-borne diseases have considerably increased the number of morbidities and mortalities over the last few decades [13, 19–25]. Despite the presence of the native primary vector, *Aedes aegypti* (Linnaeus, 1762) (Diptera: Culicidae), *Ae. albopictus* played a crucial role in the chikungunya outbreak in western DRC in 2019 [20, 21]. This outbreak was caused by the chikungunya E1-A226V strain, and *Ae. albopictus* transmits this virus more efficiently than the other *Aedes* species [21]. Recent entomological surveys revealed that *Ae. albopictus* has become established throughout the western region of the DRC and is now the most dominant *Aedes* species in the region [6, 20, 21, 26, 27]. These findings raise a serious concern that this invasive mosquito may further expand its distribution toward the inland and induce outbreaks by replacing the native vectors in newly invaded areas [20, 21, 28].

Information about *Ae. albopictus* dispersal is still scarce in the DRC. The nationwide distribution has never been studied, and its dominancy over the native *Aedes* species is unknown outside the western region [27]. Global distribution models, based on environmental variables, indicated that nearly all areas of the DRC are suitable for *Ae. albopictus* establishment [2, 29, 30]. However, these models were constructed without entomological data from the DRC, and thus the provided information was too coarse to identify high-risk areas of *Aedes*-transmitted diseases within this country. In the present study, the current distribution of *Ae. albopictus* in the DRC was determined, and the important environmental variables shaping its distribution were revealed. Based on these environmental variables, the future geographical expansion of this species was predicted. Additionally, the dominance of *Ae. albopictus* over the native *Aedes* vector species was revealed.

## Methods

### Study areas

**Fig. 1:**
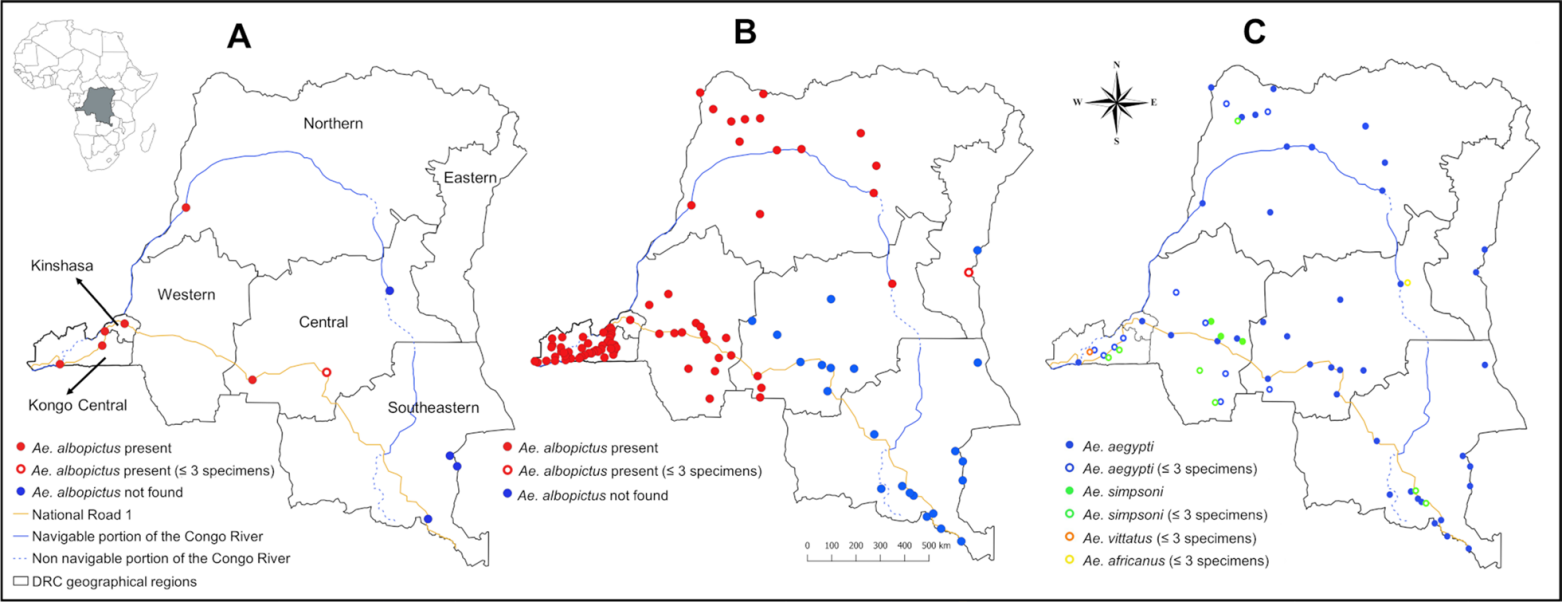
Occurrence of *Aedes* species at sampling sites (city level) in the Democratic Republic of the Congo (DRC). **A** Occurrence of *Ae. albopictus* in 2017–2019. **B** Occurrence of *Ae. albopictus* in 2022. **C** Occurrence of native *Aedes* species in 2017–2022

The DRC, the largest country in sub-Saharan Africa, spans approximately 2.4 million km^2^ and boasts diverse landscapes and climates. Prior to 1960, the country was divided into six provinces: Equateur and Orientale in the north, Léopoldville in the west, Kasai in the center, Kivu in the east, and Katanga in the southeast (Additional file 1: Figure S1). Recognizing that the division was primarily characterized by landscape and climate, it was adapted for the present study [31]. Eventually, the provinces were grouped into five regions (western, northern, central, eastern, and southeastern), thus merging Equateur and Orientale together because of the similarities in their landscape and climate (Fig. 1, Additional file 2: Figure S2). Additionally, the sizes of the central and eastern provinces were adjusted based on their landscape and climate features.

The western region features a coastal plain with hills and plateaus in the south. Savannah dominates the landscape, but tropical forests are also found in the western and northern areas. The tropical humid climate includes a 3-month dry season. This region encompasses Kinshasa and Kongo Central Province, where chikungunya outbreaks have occurred [13, 20–24]. The northern region is largely occupied by the Congo Basin and equatorial forest. This region exhibits an equatorial climate, characterized by the lack of a distinct dry season. In the northernmost part of this region, savannahs are prevalent, and the climate transitions to a tropical humid climate with the 3-month dry season. In the central region, equatorial forests cover the north, while the south features plateaus with savannahs and steppe-like vegetation. A dry tropical climate with a 2-month dry season is typical. The eastern region, marked by high hills and mountains, is characterized by lush mountain forests and a temperate mountain climate without a distinct dry season. The southeastern region is dominated by high plateaus featuring savannahs and steppe-like vegetation, with a dry tropical climate characterized by a 5-month dry season [31].

### Distribution of *Aedes* mosquitoes

Two entomological surveys were conducted: the first from May 2017 to September 2019 and the second from March to August 2022. Although the first survey covered four regions (western, northern, central, and southeastern), the focus was on Kinshasa and Kongo Central Province in the western region, where *Ae. albopictus* had been previously reported [6, 20, 25, 26]. The survey included 14 sites in Kinshasa and 9 sites in 3 cities (including the provincial capital city, Matadi) in Kongo Central Province. Since human-mediated dispersal of *Ae. albopictus* was an immediate concern, the first survey also included nine sites along the major transportation routes (Congo River and National Road 1) in the northern, central, and southeastern regions. Overall, the first survey comprised 32 sites in 11 cities (Fig. 1A, Additional file 3: Dataset S1).

The second survey covered more sites and a wider area, including the eastern region. The number of sites was increased to 24 in Kinshasa and 91 sites across 47 cities of the Kongo Central Province. In the eastern part of the western region and in other regions, samplings were conducted not only at sites along the major transportation routes but also at sites away from them, resulting in a total of 193 sites across 56 cities. Overall, the second survey comprised 308 sites in 104 cities (Fig. 1B, Additional file 4: Dataset S2). During the second survey, 31 of the 32 sites from the first survey were revisited. One site in the central region was not revisited because of weather conditions.

**Fig. 2:**
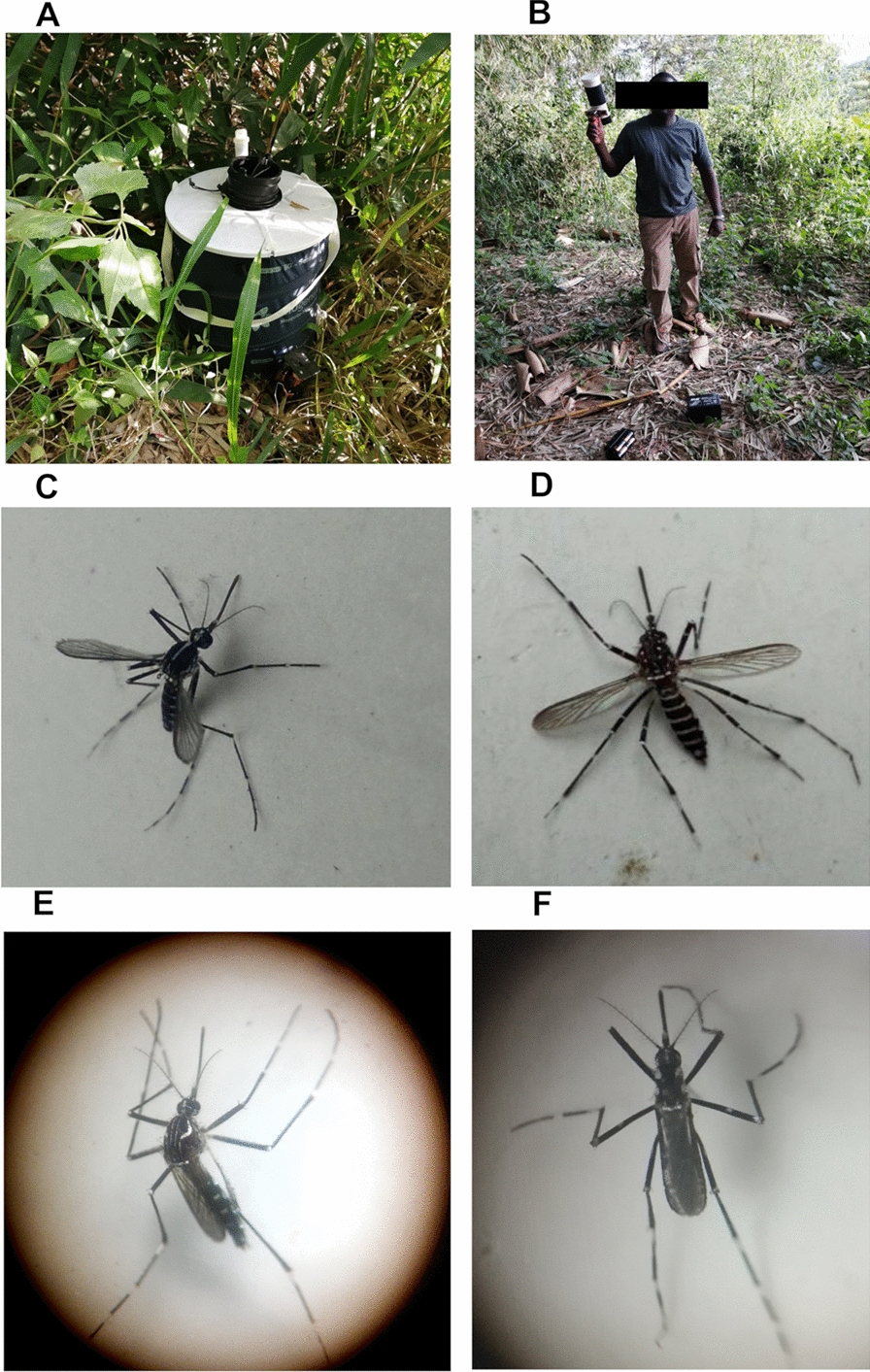
Sampling equipment and *Aedes* species specimens. **A** BG sentinel trap. **B** Prokopack electrical aspirator. **C** Female *Aedes albopictus*. **D** Female *Aedes vittatus*. **E** Female *Aedes aegypti*. **F** Female *Aedes simpsoni*

Within each site, the focus was on ecologically suitable habitats for sampling adult mosquitoes, especially around dwellings and public areas where people frequently experience daytime mosquito bites [32]. Mosquitoes were sampled between 3 and 6 p.m. using BG-Sentinel traps baited with BG-lure (Biogents Inc., Regensburg, Germany), electric aspirators (Prokopack Aspirator: John W. Hock, Gainesville, FL, USA), and mouth aspirators (Fig. 2A, B). Sampling was conducted for 3 to 7 consecutive days at each site during the first survey. In the second survey, the sampling duration was reduced to 3 days per site. To facilitate the morphological identification of adult mosquitoes, electric aspirators were run in 3-min collection waves, with the collection cups being changed after each wave to preserve the mosquito scale patterns. Additionally, the use of the electric aspirator was avoided during wet conditions, such as after heavy rain.

The collected mosquitoes were morphologically identified to the species level using Huang's identification keys [33]. Identification was conducted twice: first, in the field immediately after capture or the following morning; second, upon gathering specimens from different sites to confirm the initial identification. Both identifications were performed using either a magnifying glass or a stereoscopic microscope. If at least one specimen was collected for any species, the site was considered positive for the species. A distribution map was created using the Quantum Geographic Information System version 3.4.13, a free and open-source software (QGIS Development Team, 2020).

### Environmental variables

A thorough review of the literature was conducted on Maximum Entropy software (MaxEnt) [34], widely recognized for modeling species distribution and consistently outperforming other software [35–39]. Based on the review, 18 environmental variables were identified, each with a permutation importance (PI) of at least 5% (Table 1) [29, 40–53]. PI quantifies how much the model’s performance decreases when the value of a variable is randomly shuffled while keeping other variables constant. A lower PI indicates a less influential variable. Selecting variables with a minimum PI of 5% ensures that these environmental variables are critical in modeling the distribution of *Ae. albopictus* using MaxEnt. This choice aligns with prior studies and established practices in the field [29, 35, 40–53].

**Table 1:** Environmental variables considered for modeling the *Aedes albopictus* distribution in DRC

Code	Variable	PI (%)	References
Bio1	Annual mean temperature		[29, 44, 51]
Bio2	Mean diurnal temperature range	42.7	[42, 46]
Bio4	Temperature seasonality		[40, 46]
Bio5	Maximum temperature of the warmest month	16.9	[40, 42, 44]
Bio6	Minimum temperature of the coldest month		[40, 44]
Bio7	Temperature annual range		[42]
Bio10	Mean temperature of the warmest quarter		[45, 47, 49, 50]
Bio11	Mean temperature of the coldest quarter		[43, 45, 47, 49, 50, 52]
Bio12	Annual precipitation		[44, 52]
Bio13	Precipitation of the wettest month		[40, 42, 44, 48]
Bio14	Precipitation of the driest month		[41, 44, 46]
Bio15	Precipitation seasonality		[40]
Bio16	Precipitation of the wettest quarter		[43]
Bio17	Precipitation of the driest quarter		[43, 47, 53]
Bio18	Precipitation of the warmest quarter		[46, 51]
DEM	Digital elevation model		[51]
NDVI	Normalized Difference Vegetation Index		[29]
EVI	Enhanced Vegetation Index	40.4	[52]
	Dry season length		[55]

*PI* permutation importance

Among these variables, 15 were bioclimatic variables obtained from the WorldClim database (http://www.worldclim.com/version2) [54]. This database provides historical climate data from 1970 to 2000 with a spatial resolution of 1 km^2^. Digital elevation model (DEM) data were obtained from SRTM imagery/USGS with a resolution of about 30 m (or 1-arc second) (https://www2.jpl.nasa.gov/srtm/). Additionally, datasets for two vegetation variables, Enhanced Vegetation Index (EVI) and Normalized Differentiation Vegetation Index (NDVI), were downloaded from Modis Vegetation Index/USGS with a resolution of 1 km^2^ (https://modis.gsfc.nasa.gov/data/dataprod/mod13.php). Alongside variables identified in the literature, we incorporated dry season lengths [55].

### Modeling

The sites where *Ae. albopictus* was detected were used for modeling with MaxEnt, along with six additional positive sites from previous studies (Additional file 5: Dataset S3; Additional file 6: Dataset S4;) [21, 56]. To prevent the model from overfitting to the western region, which had a higher number of sites, 60 of the 143 positive sites in that region were randomly selected. In total, 112 positive sites were utilized to model suitable areas for *Ae. albopictus* establishment in the DRC.

Generally, environmental variables exhibit a high degree of correlation. Using these highly correlated variables in our model could lead to misleading outcomes. To prevent multicollinearity and enhance the robustness and accuracy of the MaxEnt model, the Spearman correlation coefficients were examined among the variables, as detailed in Additional file 7: Table S1 [41, 45]. A cutoff of 0.75 for these coefficients was chosen, aligning with precedents set in previous studies modeling the distribution of *Ae. albopictus* using MaxEnt (Table 1) [29, 40–52]. When a coefficient exceeded this threshold, the more important variable, as suggested by these past studies, was retained. Of the selected variables, high correlations were found in three sets and one pair of variables (Additional file 7: Table S1). One of the largest sets of variables contained six variables: annual mean temperature (Bio1), minimum temperature of the coldest month (Bio6), temperature annual range (Bio7), temperature of the warmest quarter (Bio10), mean temperature of the coldest quarter (Bio11), and digital elevation model (DEM). Another set included temperature seasonality (Bio 4), annual precipitation (Bio12), precipitation of the driest month (Bio14), precipitation seasonality (Bio15), precipitation of the driest quarter (Bio17), and dry season. The remaining set constituted annual mean temperature (Bio1), mean diurnal temperature range (Bio2), temperature of the warmest quarter (Bio10), and DEM. Additionally, EVI and NDVI were found to be highly correlated.

One variable was selected from each set, as well as from the pair [29, 40–52]. As a result, an initial full model was constructed comprising eight variables: mean diurnal temperature range (Bio2), maximum temperature of the warmest month (Bio 5), minimum temperature of the coldest month (Bio 6), precipitation of the wettest month (Bio 13), precipitation of the driest month (Bio 14), precipitation of the wettest quarter (Bio 16), precipitation of the warmest quarter (Bio 18), and EVI. Subsequently, the model was refined by excluding variables from the initial model that had a PI < 5%. For the reduced model, the hinge feature, which improves model performance and produces smoother response curves, was exclusively utilized [35, 57, 58]. The regularization multiplier was set at 2, and the sample radius was kept at 1 km to prevent overfitting. To ensure robustness, the reduced model was run ten times with cross-validation, and the median of the outputs from these ten replications was obtained [58]. The optimal model selected three variables: mean diurnal temperature range (Bio2), maximum temperature of the warmest month (Bio 5), and EVI.

The cumulative output format, which generates suitability values ranging from 0 to 100, was used to estimate the environmentally suitable areas for *Ae. albopictus* [35]. The identification of important variables for *Ae. albopictus* distribution in the DRC relied on the PI from the reduced model. Additionally, response curves were employed to assess how the model changes with the permutation of each variable individually. The Jackknife test identified both the variable that provided the most useful information and the variable that contributed the most unique information not found in other variables. The accuracy of the model was evaluated using the area under the curve (AUC). The model was deemed acceptable when the area under the curve (AUC) value was > 0.75 [58]. The median output from the reduced model was used to generate an environmental suitability map for *Ae. albopictus* in the DRC.

### Statistical analysis

Statistical analyses were conducted using the R package version 4.3.1 (R Core Team, Vienna, Austria) at a significance level of α = 0.05. The Wilcoxon Mann-Whitney test was used for comparing each environmental variable between positive and negative sites of *Ae. albopictus*. Each collection site was classified as urban or non-urban based on the categorization provided by the respective health area offices. Using this classification, the proportions of *Ae. albopictus* positive sites between urban and non-urban areas were compared using log-binomial regression. Additionally, quasi-binomial regression was employed to compare the individual-level proportions of *Ae. albopictus* and *Ae. aegypti* at the sampling sites in Kinshasa. Comparisons of the individual-level proportions of *Ae. albopictus* and *Ae. aegypti* were also conducted in both the northern and central regions.

## Results

### Distribution of *Aedes* mosquitoes

In the first survey, a total of 2842 *Aedes* mosquitoes were collected from 32 sites. Of these, 2331 (82%) were *Aedes albopictus*, 510 (18%) were *Ae. aegypti*, and 1 (< 1%) was *Aedes africanus* (Theobald, 1901) (Diptera: Culicidae) (Table 2, Additional file 3: Dataset S1). In the second survey, a total of 6751 *Aedes* mosquitoes were collected from 308 sites. *Aedes albopictus* was again the most abundant (4732; 70%), followed by *Ae. aegypti* (1909; 28%). The second survey also recorded 109 (2%) *Aedes simpsoni* (Theobald, 1905) (Diptera: Culicidae) and 1 *Ae. vittatus* (Bigot, 1861) (Diptera: Culicidae) (< 1%) (Table 2, Additional file 4: Dataset S2).

**Table 2:** Number (%) of positive sites and number (%) of samples for each species in each region

Species	Western		Northen		Central		Eastern		Southeastern		Total	
	Positive site	Sample	Positive site	Sample	Positive site	Sample	Positive site	Sample	Positive site	Sample	Positive site	Sample
Years 2017–2019												
* Aedes albopictus*	21 (91.3)	2239 (85.4)	2 (66.6)	23 (47.9)	2 (66.6)	36 (34.6)	NA	NA	0 (0)	0 (0)	25 (78.1)	2331 (82)
* Aedes aegypti*	16 (69.5)	380 (14.5)	2 (66.6)	25 (52)	3 (100)	67 (64.4)	NA	NA	3 (100)	71 (100)	24 (75)	510 (17.9)
* Aedes simpsoni*	0 (0)	0 (0)	0 (0)	0 (0)	0 (0)	0 (0)	NA	NA	0 (0)	0 (0)	0 (0)	0 (0)
* Aedes vittatus*	0 (0)	0 (0)	0 (0)	0 (0)	0 (0)	0 (0)	NA	NA	0 (0)	0 (0)	0 (0)	0 (0)
* Aedes africanus*	0 (0)	0 (0)	0 (0)	0 (0)	1 (33.3)	1 (< 1)	NA	NA	0 (0)	0 (0)	1 (< 1)	1 (< 1)
Total	23 (100)	2,619 (100)	3 (100)	48 (100)	3 (100)	104 (100)	NA	NA	3 (100)	71 (100)	32 (100)	2842 (100)
Year 2022												
* Aedes albopictus*	143 (97.9)	3,921 (82.4)	37 (100)	652 (71.4)	12 (21.4)	156 (26.2)	1 (9.9)	3 (76.9)	0 (0)	0 (0)	193 (62.6)	4732 (70)
* Aedes aegypti*	37 (25.3)	727 (15.2)	33 (89.1)	260 (28.4)	33 (58.9)	438 (73.7)	4 (36.6)	36 (92.3)	49 (84.4)	448 (99.5)	156 (50.6)	1909 (28.2)
* Aedes simpsoni*	13 (8.9)	106 (2.2)	1 (2.7)	1 (< 1)	0 (0)	0 (0)	0 (0)	0 (0)	2 (3.5)	2 (< 1)	16 (5.1)	109 (1.6)
* Aedes vittatus*	1 (< 1)	1 (< 1)	0 (0)	0 (0)	0 (0)	0 (0)	0 (0)	0 (0)	0 (0)	0 (0)	1 (< 1)	1 (< 1)
* Aedes africanus*	0 (0)	0 (0)	0 (0)	0 (0)	0 (0)	0 (0)	0 (0)	0 (0)	0 (0)	0 (0)	0 (0)	0 (0)
Total	146 (100)	4,755 (100)	37 (100)	913 (100)	56 (100)	594 (100)	11 (100)	39 (100)	56 (100)	450 (100)	308 (100)	6751 (100)

*NA* Not applicable

*Aedes albopictus* was identified at 25 (78%) of 32 sites in the first survey and at 193 (62%) of 308 sites in the second survey. In the western region, *Ae. albopictus* was collected at over 90% of the sites in both surveys. Within Kinshasa, *Ae. albopictus* was found at 12 (91%) of 14 sites in the first survey and at 21 (87%) of 24 sites in the second survey. All of the negative sites were in the urban areas of the northern part of Kinshasa. In Kongo Central Province, *Ae. albopictus* was collected at all 9 (100%) sites in the first survey and 91 (100%) sites in the second survey. In the northern region, *Ae. albopictus* was collected at 2 (66%) of 3 sites during the first survey and at all 37 (100%) sites during the second survey. One previously negative site in the region became positive. In the central region, *Ae. albopictus* was collected at 2 (66%) of 3 sites during the first survey and at 12 (21%) of 56 sites during the second survey. However, the second survey failed to collect this mosquito in Bupole in Mbuji-Mayi City, where it was present in the first survey (Additional file 3: Dataset S1, Additional file 4: Dataset S2). Moving to the eastern region, *Ae. albopictus* was found at only 1 (9%) of 11 sites. In the southeastern region, despite visiting 3 sites in the first survey and 58 sites in the second survey, *Ae. albopictus* was not found.

In the first survey, 94% (30 sites) of all the sampling sites were classified as urban, while the proportion of urban sites was 46% (142 sites) in the second survey. When the data from the second survey were analyzed, the proportions of positive sites for *Ae. albopictus* were 67% and 59% for urban and non-urban areas, respectively. However, the difference between these proportions was not statistically significant with a log-binomial regression (presence ratio: 1.13, 95% CI: 0.95–1.34).

*Aedes aegypti* was identified in all the regions (Fig. 1C, Table 2). This species was found at 24 (75%) of 32 sites in the first survey and at 156 (51%) of 308 sites in the second survey. While the proportion of positive sites was > 80% in the northern and southeastern regions in the second survey, it was found at only 25% of the sites in the western region. Notably, there was a stark contrast in this region: *Ae. aegypti* was present at almost all sites in Kinshasa (22 of 24 sites) but only at 4% of the sites in Kongo Central (4 of 91 sites) during the second survey.

In the first survey, both *Ae. aegypti* and *Ae. albopictus* were detected together at 28% of all sites. Specifically, they co-occurred at 64% (9 of 14 sites) in Kinshasa and 55% (5 of 9 sites) in Kongo Central in the western region (Additional file 3: Dataset S1). In the second survey, the co-occurrence was found at 24% of all sites (Additional file 4: Dataset S2, Additional file 8: Figure S3). They were found at 79% (19 of 24) of sites in Kinshasa. However, *Ae. aegypti* was present at the sites in the most urbanized area of the town, where *Ae. albopictus* was not found. Although the individual level proportions of *Ae. albopictus* and *Ae. aegypti* in Kinshasa were 62% and 38%, respectively, the difference was not statistically significant (quasibinomial regression, proportion ratio: 1.6, 95% CI: 0.99—2.81). The two species shared only 15 (14%) of 106 sites in the other part of the western region. When all of the sites in the western region were considered, the individual level proportions of *Ae. albopictus* and *Ae. aegypti* samples were 84% and 16%, respectively. The difference was statistically significant (quasibinomial regression, proportion ratio: 5.4, 95% CI: 4.99—5.84).

Both *Ae. albopictus* and *Ae. aegypti* were found together at most of the sites (89%) in the northern region during the second survey, but the individual level proportion of *Ae. albopictus* was significantly greater than that of *Ae. aegypti* (quasibinomial regression, proportion ratio: 2.5, 95% CI: 2.17—2.90). However, the proportion of *Ae. albopictus* was lower in the central region (quasibinomial regression, proportion ratio: 0.36, 95% CI: 0.30—0.43) (Additional file 4: Dataset, Additional file 8: Figure S3).

*Aedes simpsoni* was detected at 16 (5%) of 308 sites in the second survey (Table 2, Additional file 4: Dataset S2). Both *Ae. simpsoni* and *Ae. albopictus* were found at 4% of all the sites. The distribution of this mosquito species was more confined within the eastern part of the western region (Fig. 1C). In this part, *Ae. simpsoni* outnumbered *Ae. albopictus* and *Ae. aegypti* in two cities, Idiofa and Kikwit (Additional file 8: Figure S3). Only one individual of *Ae. africanus* was collected in the central region during the first survey, and one individual of *Ae. vittatus* was collected in the western region during the second survey (Fig. 1C, Table 2).

### Environmental suitability

**Fig. 3:**
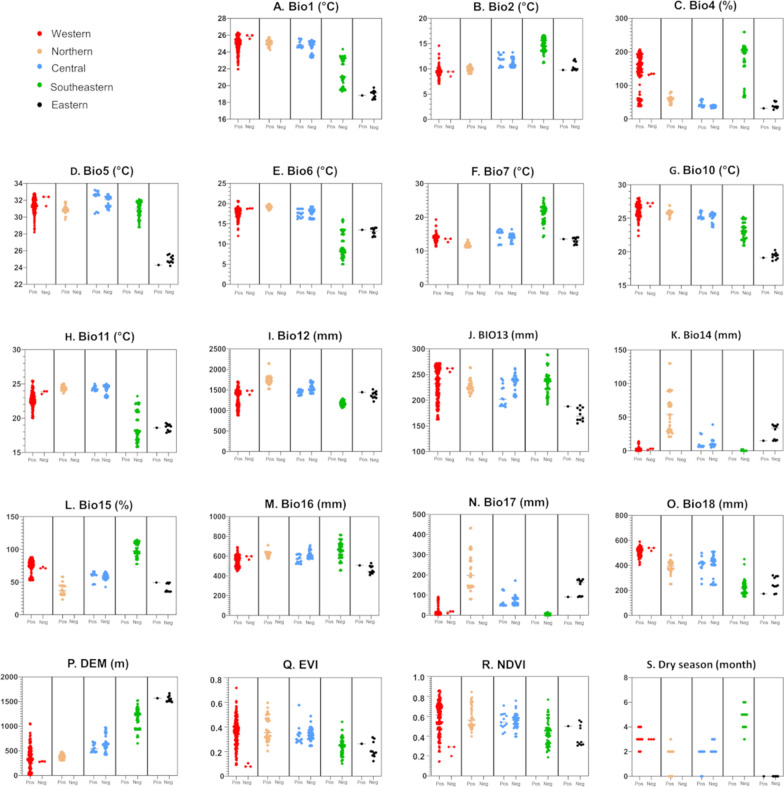
Distributions of environmental variables at positive and negative sites of *Aedes albopictus* in the five regions. Black horizontal bars indicate the medians. DEM, Digital Elevation Model; EVI, Enhanced Vegetation Index; NDVI, Normalized Difference Vegetation Index

**Fig. 4:**
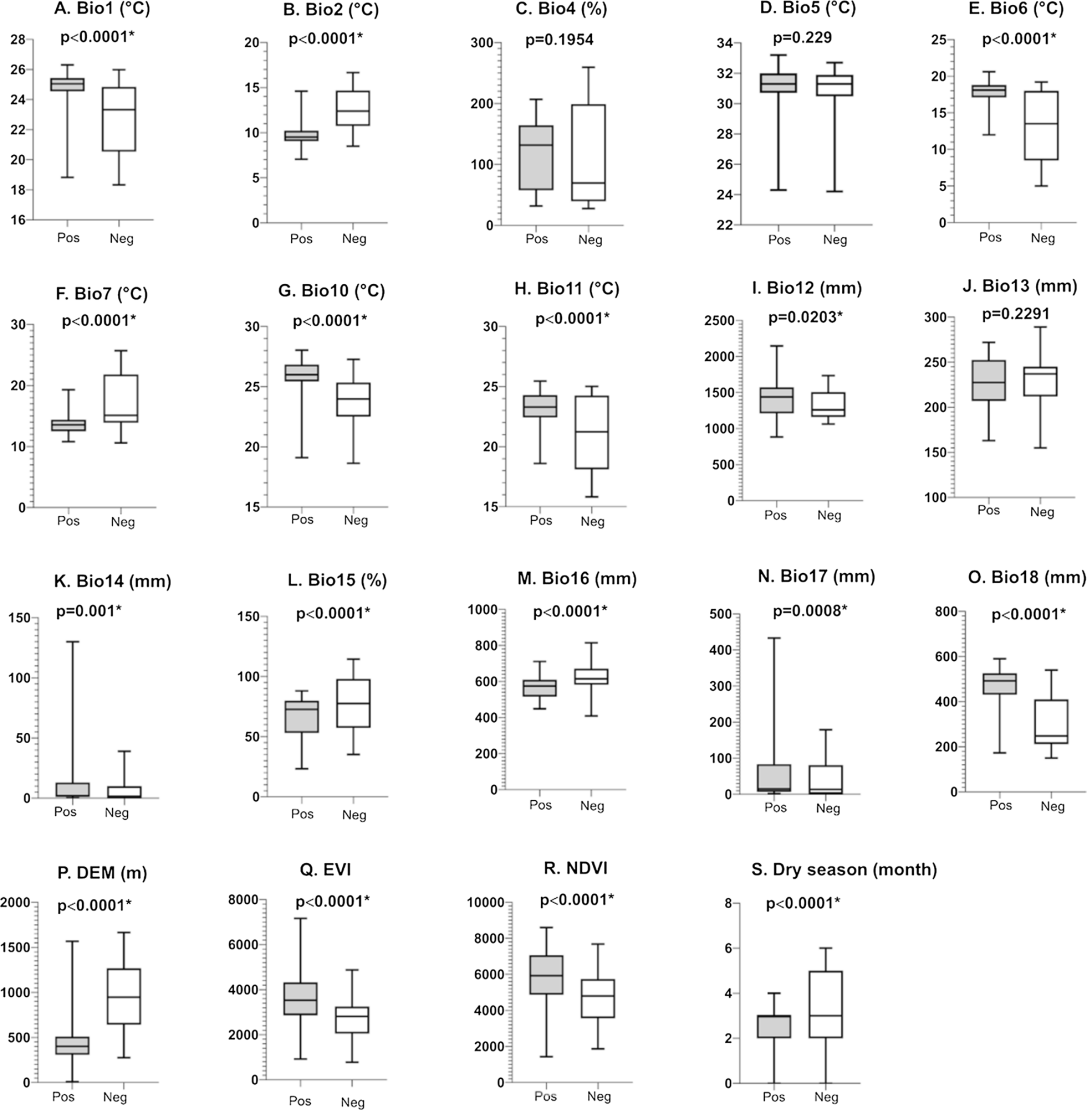
Comparisons of environmental variables between the positive and negative sites of *Aedes albopictus* sites. Each panel shows box plots of the first quartile, median, third quartile, and minimum and maximum values in *Aedes albopictus*-positive and -negative sites. DEM, Digital Elevation Model; EVI, Enhanced Vegetation Index; NDVI, Normalized Difference Vegetation Index

Based on environmental data obtained for each site, the western region was characterized by high temperature seasonality (Fig. 3C), low precipitation during the driest month (Fig. 3K), and low elevation (Fig. 3P). The northern region was characterized by high precipitation throughout the year (Fig. 3I, J, K, M, N, O) and low elevation (Fig. 3P). The southeastern region had high temperature and precipitation seasonality (Fig. 3C, L), high temperature ranges (Fig. 3B, F), and high elevation (Fig. 3P) but also had low temperature (Fig. 3A, E, H) and a severe dry season (Fig. 3K, N, S). The eastern region had low temperatures (Fig. 3A, D, H) and high elevation (Fig. 3P) (Additional file 9: Dataset S5). In the central region, the values of all variables fell within the ranges observed in the other regions (Fig. 3A-S). The Wilcoxon-Mann-Whitney tests revealed that of the 19 variables, 16 were significantly different between *Ae. albopictus* positive and negative sites (Fig. 4). The negative sites had colder climate (Fig. 4A, E, G, H), greater temperature ranges (Fig. 4B, F), higher elevation (Fig. 4P), lower Vegetation Index (Fig. 4Q, R), and longer and drier dry seasons (Fig. 4K, N, S) compared to the positive sites.

**Fig. 5:**
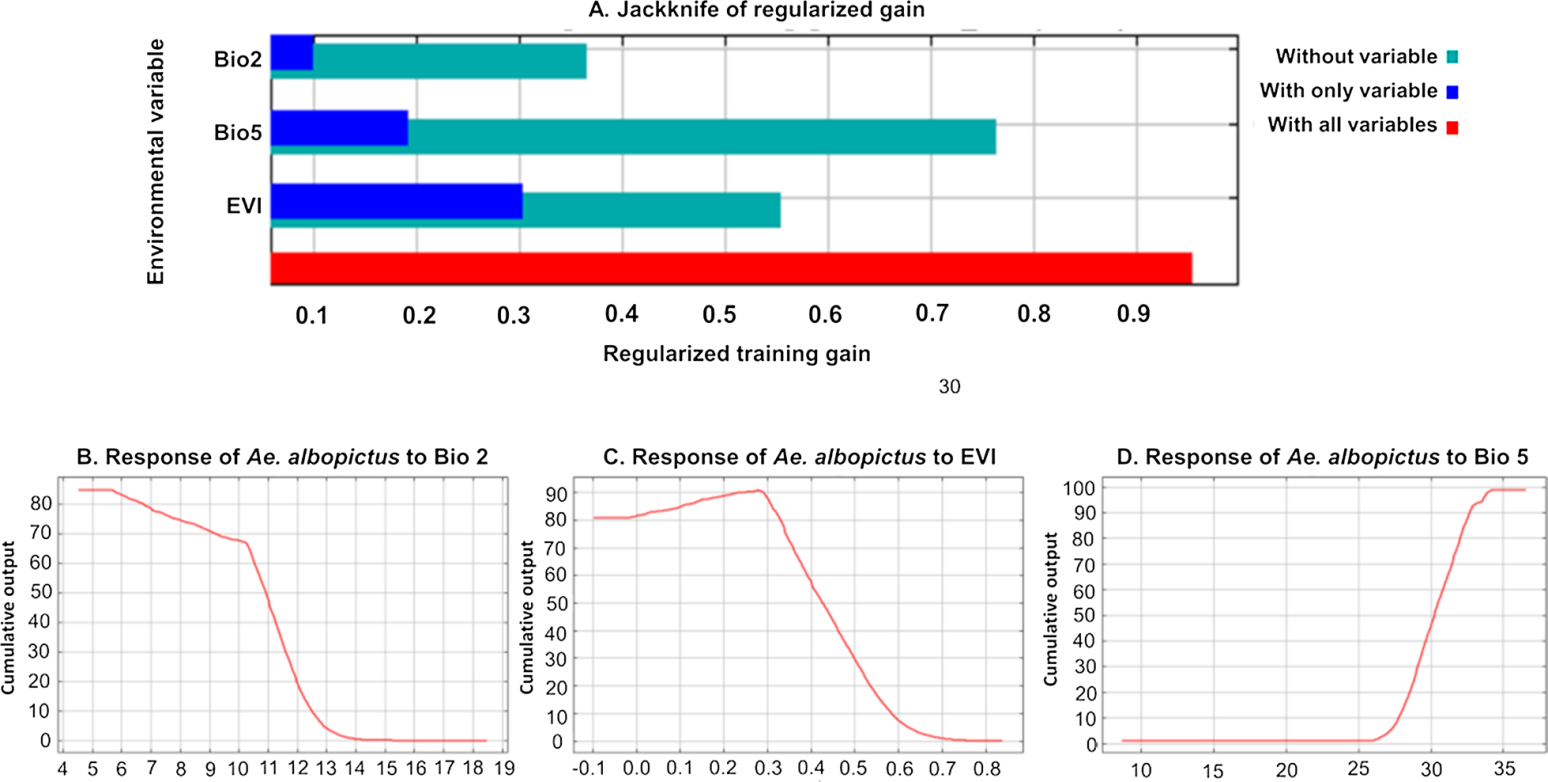
Jackknife plot and response curves for *Aedes albopictus* suitability. **A** Jackknife plot in relation to mean diurnal temperature range, maximum temperature of the warmest month, and Enhanced Vegetation Index. The jackknife plot reflected the impact of each variable on the entire model. Light blue indicates the impact on the model when this variable is excluded, and dark blue indicates the independent contribution of this variable to the model. **B** Response curve in relation to the mean diurnal temperature range. **C** Response curve in relation to the Enhanced Vegetation Index. **D** Response curve in relation to the maximum temperature of the warmest month. The curves show how each environmental variable affects the MaxEnt prediction. The red line is the mean response of the ten MaxEnt replications. *EVI* Enhanced Vegetation Index)

The AUC of the optimal model was 0.871 ± 0.036, and the mean diurnal temperature range (Bio 2) had the highest PI (42.7%), followed by EVI (40.4%) (Table 1). The jackknife plot also revealed that the mean diurnal temperature range (Bio2) contributed the most distinctive information to the model, while EVI alone provided the most useful information. The response curves indicated that the most suitable area for *Ae. albopictus* was predicted to have a mean diurnal temperature range (Bio 2) < 10 °C, an EVI between 0.2 and 0.3, and a maximum temperature of the warmest month (Bio 5) > 34.2 °C (Fig. 5).

**Fig. 6:**
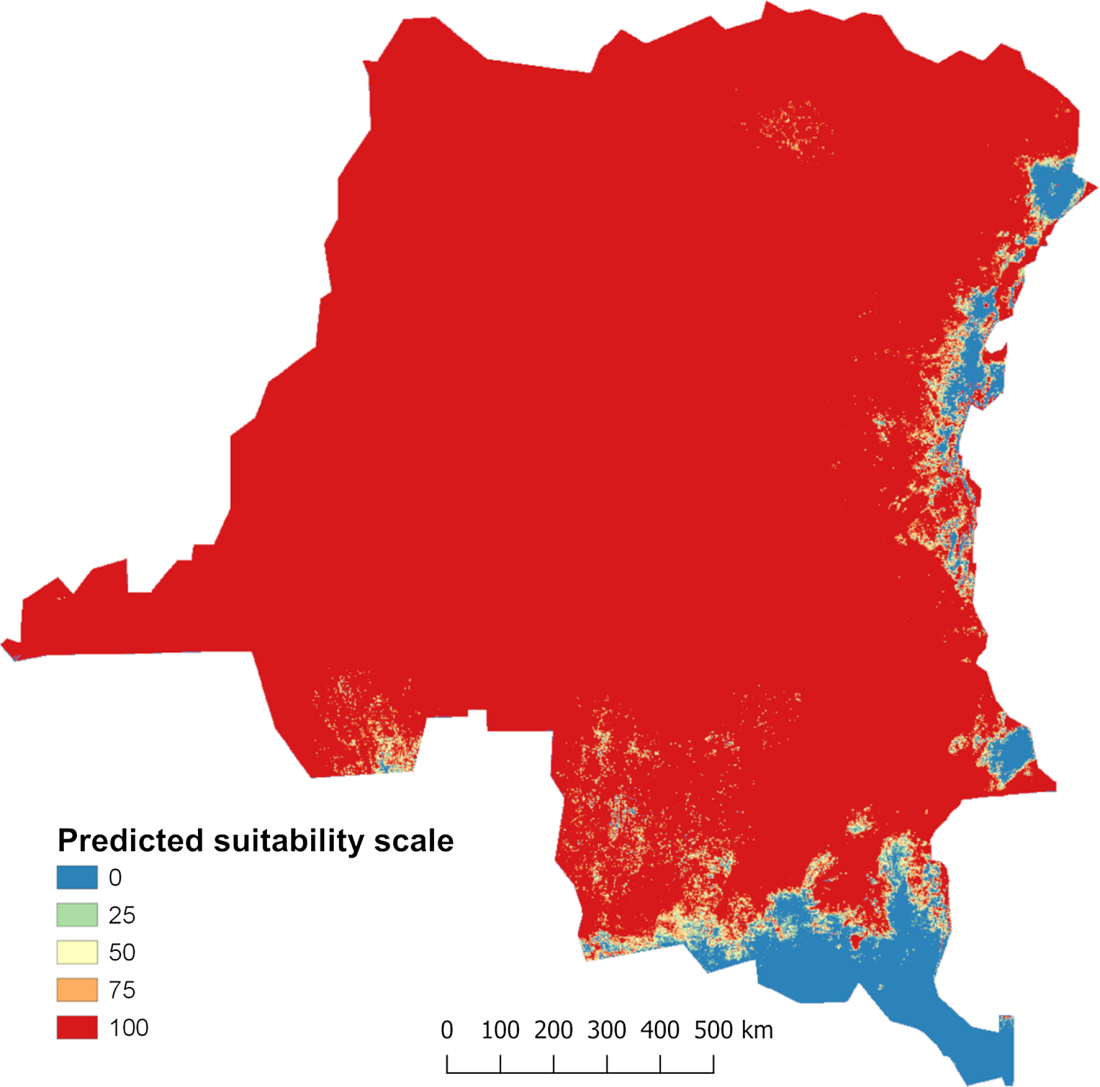
Suitable areas for *Aedes albopictus* establishment predicted by the optimal MaxEnt model

The MaxEnt model predicted that most of the DRC is suitable for the establishment of *Ae. albopictus* (Fig. 6). The western, central, and northern regions were forecasted to be highly suitable with *Ae. Albopictus*, except from a small area in the southern part of the western region where the suitability was low. In the eastern and the southeastern regions, large areas were unsuitable. The model successfully predicted all the positive sites within highly suitable areas, with the exception of the single positive site in the eastern region that fell in a low-suitable area. Additionally, negative sites from the western, central, and the northern part of the southeastern region were predicted to be highly suitable. However, negative sites in the southern part of the southeastern region and in the eastern region were in areas projected to be less suitable or unsuitable for *Ae. albopictus*.

## Discussion

The present study confirmed the expansion of *Ae. albopictus* distribution to the western, northern, and central regions of the DRC. The MaxEnt model predicted that these regions are suitable for the establishment of *Ae. albopictus*. Prior to our study, *Ae. albopictus* was first reported in the DRC in 2016 in Kinshasa in the western region [6]. In 2019, this mosquito was found in other parts (Kasangulu and Matadi) of the western region [20, 21, 27]. A study conducted in 2021 reported the presence of *Ae. albopictus* in Boende in the northern region [56]. However, our first survey demonstrated that by 2019 this mosquito was already in the northern and the central regions. *Aedes albopictus* was found in almost all the sampling sites in the western and northern regions, except for a few urban sites in Kinshasa. The results suggest that the mosquito population has stabilized in these regions, which, because of their climatic characteristics, offer favorable breeding grounds for *Ae. albopictus* [2]. *Aedes albopictus* has also become established in urban settlements in other central African countries [8, 10, 18, 59–62]. However, *Ae. albopictus* is known to prefer areas with abundant vegetation within urban and peri-urban settlements [60, 62–65]. Since the sites without *Ae. albopictus* in Kinshasa had the lowest EVI values, this result is consistent with the previous findings (Additional file 9: Dataset S5).

In the central region, most positive sites are connected to those in the western and northern regions through the National Road 1 and the Congo River, respectively. In 2019, a few specimens of *Ae. albopictus* were collected in Mbuji-Mayi, located in the central part of the region; however, this species was not found there in 2022 (Fig. 1A, B). These results suggest that *Ae. albopictus* is spreading in the central region through these transportation routes, although the mosquito is not well established. The MaxEnt species distribution model predicts that the entire central region is suitable for the establishment of *Ae. albopictus*, indicating the potential for this mosquito to establish a stable population in the region in the near future.

Finding *Ae. albopictus* in the eastern region was unexpected, considering its isolation from the western and northern regions due to distance and poor road conditions. Frequent air travel between the area and Kinshasa might have introduced *Ae. albopictus* [66]. Since a small number of *Ae. albopictus* was collected at one site only, it might not yet have established a stable population in the region. Since a large part of the eastern region is located > 2000 m above sea level, and the highland area includes mountains up to 5000 m [31], the harsh environment may prevent *Ae. albopictus* from establishing a stable population in the highlands [67].

*Aedes albopictus* was not found in the southeastern region, which includes Lubumbashi, the second largest city in the DRC. Although the road traffic from the western and central regions is not significant, intense air traffic between Kinshasa and Lubumbashi suggests the potential introduction of this mosquito species to the area in the near future. However, the climate may likely limit the introduction of this mosquito in the region. The MaxEnt model predicts that the southern part of this region is unsuitable for the establishment of *Ae. albopictus*. The response curve suggests that the mean diurnal temperature range (Bio 2) in the suitable area should be < 10 °C; however, the collection sites in the southern part of the region had a mean diurnal temperature range > 10 °C. The statistical test also revealed that the mean diurnal temperature range was significantly lower in the positive sites. Moreover, the mean diurnal temperature range was negatively correlated with the annual mean temperature, and the negative sites had a significantly lower annual temperature. The immature stages of mosquitoes require specific temperature conditions for successful development [2, 45]. Large diurnal temperature variations, resulting in varying temperatures experienced by these life stages, can potentially have a negative influence on their growth rates and development times, while warmer temperatures in the optimal range positively accelerate their life cycle [2, 45, 67–70]. The greater diurnal temperature range and low temperature may likely limit the development and survivorship of *Ae. albopictus* in the southern part of the southeastern region. Additionally, the area experiences a prolonged dry season lasting up to 6 months, which would pose a challenge for the establishment of *Ae. albopictus* [68]. The statistical test showed that the negative sites had a significantly longer dry season. The dry climate reduces the availability of mosquito breeding sites, and further their development and survivorship.

Nevertheless, *Ae. albopictus* exhibits a strong physiological and ecological plasticity that allows it to adapt to a wide range of environmental conditions [68, 69]. This species occurs in the temperate areas where the temperatures are considerably lower than those in the southeastern region [2, 69, 71]. For instance, in La Reunion, *Ae. albopictus* has established in the highland area with cooler climate [71]. Considering that *Ae. albopictus* in temperate regions produces diapause eggs for overwintering, it is essential to genetically identify the origin of the introduced population in the DRC [67, 68]. Also, *Ae. albopictus* inhabits areas of Madagascar where the dry season is > 6 months [59]. Eggs of *Ae. albopictus* are known to be drought resistant [55, 69]. On the other hand, the MaxEnt model predicted that the northern part of the southeastern region is suitable for this mosquito. Therefore, it is crucial to continuously monitor the southeastern region for this species by implementing a surveillance system, which should also be applied to other regions.

During the yellow fever outbreak in the western part of the western region in 1928, *Ae. aegypti* was the predominant vector species [72]. In 1993, *Ae. aegypti* was still predominant in the urban and semi-rural areas of the western part, followed by *Ae. simpsoni*, *Aedes argenteopunctatus*, and *Ae. vittatus* [73]. However, during the 2019 chikungunya outbreak, *Ae. albopictus* was almost the only vector found in most of the affected areas of the western part. Even though a substantial number of *Ae. aegypti* was recorded in Kinshasa during the outbreak, *Ae. albopictus* was still the most abundant species [20, 21]. Nevertheless, in certain sites in the eastern part of the western region in the present study, *Ae. simpsoni* outnumbered both *Ae. aegypti* and *Ae. albopictus*. A further study is needed to reveal the factors that could explain the abundance of *Ae. simpsoni*.

During a yellow fever outbreak in Gemena City in the northern region in 1962, 90% of the *Aedes* mosquitoes collected were *Ae. simpsoni*, while the remaining 10% were *Ae. aegypti* [74]. In the present study, *Ae. albopictus* was predominant in the same city, followed by *Ae. aegypti*, and *Ae. simpsoni* was < 1% of the sampled mosquitoes (Additional file 4: Dataset 2). These results suggest a rapid replacement of native *Aedes* species by *Ae. albopictus* in the western and northern regions. Similar trends have been observed in central Africa, where studies indicate that *Ae. albopictus* becomes the predominant species in suburban areas, while *Ae. aegypti* remains predominant in highly urbanized regions [59–62].

The studies in central Africa also suggested that the introduced *Ae. albopictus* population affected the epidemiology of chikungunya and dengue despite the presence of the native vector, *Ae. aegypti* [18, 69]. Most reports of chikungunya and dengue in the DRC occurred after the initial detection of *Ae. albopictus* in central Africa in 2000 [5, 13, 19]. Although *Ae. albopictus* was first reported in the DRC in 2016, the species might had been introduced to the country in the late 1990s and contributed to the first chikungunya outbreak in Kinshasa in 1999 [6, 13]. Chikungunya and dengue cases have mainly been reported in the western and the northern regions, where *Ae. albopictus* has become the predominant species [19]. Moreover, the chikungunya E1-A226V strain was detected in the 2019 chikungunya outbreak in the western region. The strain is known to be more effectively transmitted by *Ae. albopictus* than the other *Aedes* species [21]. Despite the increasing burden of chikungunya and dengue in the DRC, there is little information on the contribution of *Ae. albopictus* in the viral transmissions, and more studies are needed.

## Limitation

This study has some limitations that should be addressed in future studies. First, due to the extensive number of sites, time constraints, and challenges in accessing some areas, the sampling effort and tools were not consistent across all the sites. Species composition and abundance of mosquitoes may vary seasonally, and seasonality varies geographically in such a large country. Although the surveys were conducted during the rainy season, the spatio-temporal variations and sampling effort might have affected the results. Importantly, these constraints limited our ability to conduct more comprehensive analyses on the interaction between *Ae. albopictus* and other species, such as *Ae. aegypti*, which are critical for vector control strategies. The environmental variables used in this study were mostly selected based on studies conducted in the temperate areas of Europe and tropical areas of South America, as there are limited studies in Central Africa. Appropriate variables for the central African context may differ. Crucially, this study did not include sampling of *Aedes* mosquito larvae, thereby missing the characterization of breeding habitats, which is pivotal for vector control. Future studies should aim to address these gaps, enhancing our understanding of vector dynamics and improving control measures in the DRC.

## Conclusions

*Aedes albopictus* has become well established in the western and northern regions of the DRC and predominates over the native *Aedes* species. This mosquito species is actively extending its distribution, gradually replacing indigenous *Aedes* species. While most of the country provides a suitable environment for the establishment of *Ae. albopictus*, exceptions include the highlands of the eastern region and the southern part of the southeastern region, where climatic conditions likely constrain its expansion. The findings of this study will serve as a foundational reference for future research and will prove invaluable to public health officials in guiding their efforts to control *Aedes*-transmitted diseases in the DRC.

### Supplementary Information

**Additional file1: Figure S1**. Administrative subdivision of the DRC before 1960.

**Additional file 2: Figure S2**. Geographical regions and sampling sites.

**Additional file 3: Dataset S1**. Original dataset for 2017-2019 survey.

**Additional file 4: Dataset S2**. Original dataset for 2022 survey.

**Additional file 5: Dataset S3**. Additional presence data.

**Additional file 6: Dataset S4**. Presence dataset used in MaxEnt.

**Additional file 7: Table S1**. Spearman correlations among environmental variables.

**Additional file 8: Figure S3**. Coexistence of *Aedes* *albopictus* and major African-native *Aedes* species. **A** Frequency of *Aedes* species presence in sampling sites. **B** Relative abundance of *Aedes* species in sampling sites.

**Additional file 9: Dataset S5**. Environmental variable values.

## Data Availability

All data generated or analysed during this study are included in this published.

## Abbreviations

AUC: Area under the curve
DEM: Digital elevation model
DRC: Democratic Republic of the Congo
EVI: Enhanced Vegetation Index
MaxEnt: Maximum entropy
NDVI: Normalized Difference Vegetation Index
PI: Permutation importance
