# Supplementary figures and images for: Geographic expansion of the introduced Aedes albopictus and other native Aedes species in the Democratic Republic of the Congo
Source: Parasit Vectors. 2024 Jan 26;17:35. doi: 10.1186/s13071-024-06137-4 (PMC10811949; doi:10.1186/s13071-024-06137-4)

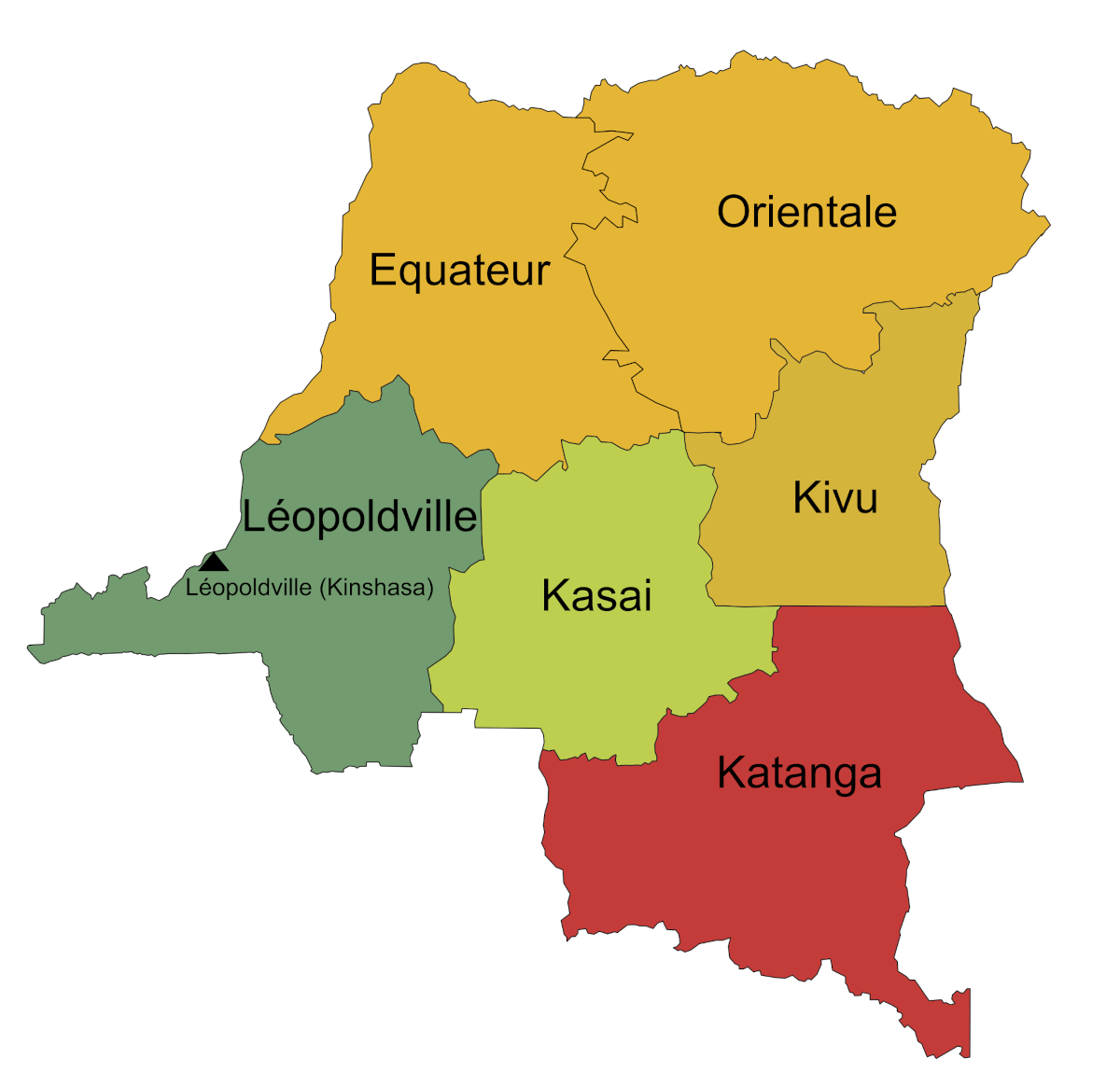

Supplement: Supplementary file 1 — Additional file1: Figure S1. Administrative subdivision of the DRC before 1960. [file 13071_2024_6137_MOESM1_ESM.tiff]

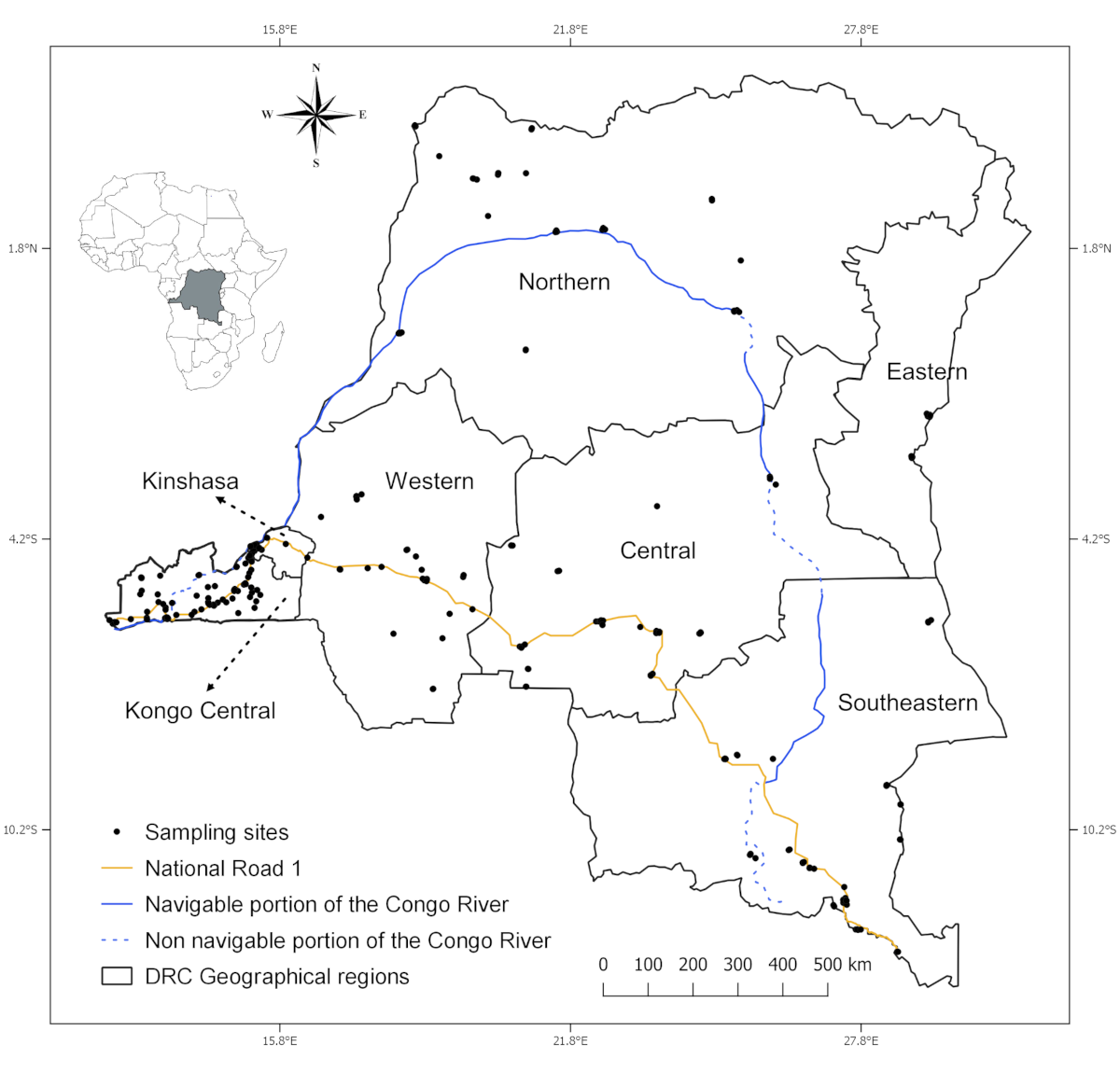

Supplement: Supplementary file 2 — Additional file 2: Figure S2. Geographical regions and sampling sites. [file 13071_2024_6137_MOESM2_ESM.tif]

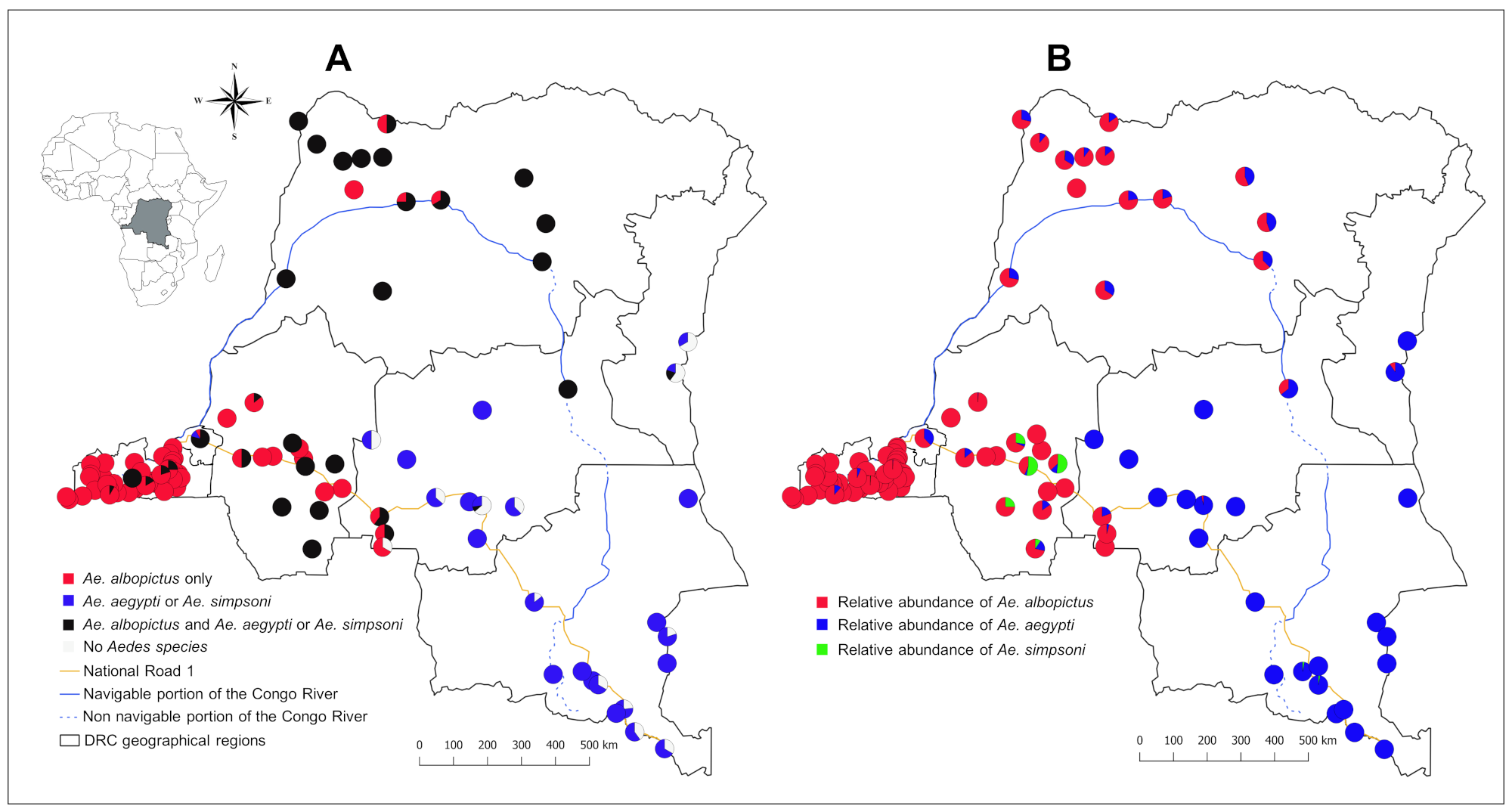

Supplement: Supplementary file 8 — Additional file 8: Figure S3. Coexistence of Aedes albopictus and major African-native Aedes species. A Frequency of Aedes species presence in sampling sites. B Relative abundance of Aedes species in sampling sites. [file 13071_2024_6137_MOESM8_ESM.tif]
